# Survey data on Vietnamese retail investors׳ trading behavior and their psychological and behavioral patterns

**DOI:** 10.1016/j.dib.2018.05.113

**Published:** 2018-05-25

**Authors:** Thuy Chung Phan, Marc Oliver Rieger, Mei Wang

**Affiliations:** a University of Trier, Chair of Banking and Finance, 54296, Germany; b University of Economics, Hochiminh City, School of Banking, 279 Nguyen Tri Phuong, District 10, Ho Chi Minh City, Vietnam; c WHU-Otto Beisheim School of Management, Chair of Behavioral Finance, 56179 Vallendar, Germany

## Abstract

The data article describes self-assessments of 621 Vietnamese retail investors on their trading behavior, psychological attributes and socio-demographic characteristics. The dataset was obtained from a randomized survey of 3144 Vietnamese participants on financial attitudes and practice that has been used in Phan et al. [5]. A supplemental material data contains the full text, codes and numerical values of survey instruments. Discussion of theoretical frameworks and the development of hypothesis and measurement of survey variables are found in the associated research article [6].

**Specifications Table**

**Table:** 

Subject area	Finance
More specific subject area	Behavioral finance/ Behavioral retail portfolios
Type of data	Table, text file
How data was acquired	Survey
Data format	Raw, filtered and partially analyzed
Experimental factors	Raw data obtained from a survey on Vietnamese subjects
	who were trading on the Vietnamese stock market. Data
	with non-response and extreme values were eliminated.
Experimental features	The experiment focuses on examining retail investors׳
	trading behavior, selected psychological and behavioral
	patterns including: overconfidence, self-control, social trans-
	mission bias, risk attitudes and time preferences.
Data source location	Vietnam
Data accessibility	Data is available with this article.

**Value of the data**

• The data provide insights on the investment behavior of retail investors in anemerging market which covers several interesting behavioral factors at once.
• The data can help to predict the trading mistakes of retail investors, particularlyover-trading and under-diversi cation by examining their psychological and behavioral biases.
• The data offer an opportunity to measure the extent to which trading mistakesretail investors made in emerging markets (in comparison to advanced marketsstudied in previous works).
• The data allow for a comparison of psychological and behavioral biases with other similar studies amongst retail investors in a meta-analysis.
• The dataset can be employed for further analysis which provides insights into financial information acquisition and its link to the trading frequency of retail investors.

## 1 Data

The attached dataset (Appendix A) contains 621 records obtained from a randomized survey on 3144 Vietnamese financial clients concerning financial attitudes and behavior which have been first analyzed in the study of Phan et al. [5]. In this paper, we only present results on their psychological, behavioral patterns and trading activities in the Vietnamese stock market. Table 1, Table 2 summarize surveyed variables of the provided dataset which can help estimate selected psychological variables (such as illusion of control, self-attribution, self-control, overconfidence in confidence interval estimates (or miscalibration), self-reported risk tolerance and time preferences), equity trading behavior and socio-demographic characteristics at the individual level. Discussion of theoretical backgrounds and development in the measurement and calculation of the survey variables can be found in the associated research article [6].

**Table 1:** Survey variables.

Code(s)	Variable(s)	Variable type	Value labels
*Panel A–Psychological variables*			
iq1–iq4	Illusion of control	Ordinal	Likert-scale where
			“1=Strongly agree”
			“5=Strongly disagree”
aq5–aq6	Self-attribution	Ordinal	Likert-scale where
			“1=Strongly agree”
			“5=Strongly disagree”
cq7–cq16	Self-control	Ordinal	Likert-scale where
			“1=Very much like me”
			“5=Not at all like me”
upper	Upper bound	Scale	As reported
average	Estimated average	Scale	As reported
lower	Lower bound	Scale	As reported
psyrisk	Willingness to take risks	Ordinal	Likert-scale where
			“0=risk averse”
			“10=risk prone”

*Panel B–Time preferences*			
q1Delay1m	1-month delay	Scale	As reported
q2Upfront1m	1-month upfront-delay	Scale	As reported
q3Delay1y	1-year-delay	Scale	As reported
q4Upfront1y	1-year-upfront-delay	Scale	As reported

*Panel C–Trading behavior*			
qA1_r	Equity investor	Nominal	1: Applicable; 0: Not
qA2_r	Investment portfolio size	Ordinal	1: < 50 Million VND
			2: 50–100 Million VND
			3: 100–250 Million VND
			4: 250–500 Million VND
			5: 500–1 Billion VND
			6: > 1 Billion VND
qA3_r	Trading frequency	Ordinal	1: 1–3 trades; 2: 4–5 trades
	(#trade/month)		3: 6–10 trades; 4: 11–15 trades
			5: > 15 trades
qA4_r	Evaluate frequency	Ordinal	1: Annually; 2: Quarterly
			3: Monthly; 4: Weekly
			5: A couple of times/week
			6: Daily; 7: > 3 times/day

**Table 2:** Survey variables (cont.).

Code(s)	Variable(s)	Variable type	Value labels
qA5a_r	Stock-market-fund investors	Nominal	1: Applicable; 0: Not
qA5b_r	The number of different single	Ordinal	0: No stocks; 1: 1 stock
	stocks in a respondent׳s current		2: 2 stocks; 3: 3–5 stocks
	investment portfolio		4: 6–10 stocks; 5:> 10 stocks
qA6a…qA6g	Reasons for stock purchases	Nominal	1: Applicable; 0: Not
qA7a….qA7g	Sources of information acquisition	Nominal	1: Applicable; 0: Not
qA8a…qA8j	Reasons for stock sales	Nominal	1: Applicable; 0: Not

*Panel D–Socio-demographic backgrounds*			
gender	Gender	Nominal	0: Male; 1: Female
age	Age	Ordinal	1: 18–35 years old
			2: 36–50 years old
			3: > 51 years old
selfedu	Education level	Ordinal	1: Less than high school
			2: High school leaving cert.
			3: Vocational school degree
			4: University degrees
unistudent	Students at university	Nominal	1: Applicable; 0: Not
selfmajor1	Specialization in the first and	Categorical	1: Economics
selfmajor2	second degrees respectively		2: Natural sciences/Medicine
			3: Social sciences/Others
employment	Currently employed	Nominal	1: Applicable; 0: Not
q15a…q15g	Occupational status	Nominal	1: Applicable; 0: Not
q16	Financial occupation	Nominal	1: Applicable; 0: Not
income	Monthly net income	Ordinal	1: 5 Million VND or less
			2: 5–10 Million VND
			3: 10–20 Million VND
			4: > 20 Million VND

## 2 Experimental design, materials and methods

### 2.1 Questionnaire design

The questionnaire consists of three parts. The first part includes self-reported responses to scales measuring various facets of overconfidence and other psychological attributes. The second part has hypothetical choice tasks which attempt to extract an individual׳s time preferences. The last part includes detailed information about participants׳ stock holdings, sources of financial information acquisition and their demographic and socio-economic characteristics.

In the first part, selected psychological variables were measured in different ways. Firstly, following Dorn and Huberman [2], six items, on a five-point-Likert-scale from 1=“totally agree” to 5=“totally disagree”, were used for extracting an individual׳s illusion of control and self-attribution biases. Secondly, ten items of the self-control scale (SCC) adapted from Tangney et al. [8] were included to assess self-control. All ten items are rated on a five-point-Likert-scale, ranging from 1=“very much like me” to 5=“not at all like me”. Thirdly, a question about investors׳ probability judgments on the future return of the Vietnam Ho Chi Minh stock index (called VN–index) were used for capturing their levels of miscalibration. Moreover, we also included a Likert-scale item measuring participants׳ willingness to take risks on a scale where 0=“risk averse” and 10=“risk-prone”, following Dohmen et al. [1].

In the second part, we included four hypothetical choice tasks to elicit an individual׳s ability to defer gratifications. Following Sutter et al. [7], we asked participants to state an early payoff which makes them indifferent with a fixed later payoff of 200 EUR (1 EUR is equivalent to approx. 10,000 VND PPP [4]) in four different time frames. In the one-month-delay task, subjects were asked to give an early payoff today for a delay in one month. For the second task, the delay in one month is kept but the early payoff is shifted one month later from today (one-month upfront-delay). Similarly, the third and fourth tasks ask subjects to differentiate a payoff between today and one year (one-year delay) and between one month and thirteen months (one-year upfront-delay). For each task, we show them an illustration of a combination between early and later payoffs as summarized in Figure 1 in Phan et al. [6]׳s paper.

In the last part, we focus on an individual investor׳s trading behavior and socio-demographic attributes. In particular, we asked participants about their trading activities such as the frequency at which they trade stocks and evaluate their investment performance, portfolio composites, sources of financial information acquisition and reasons for their stock purchases and sales. Moreover, questions about age, gender, educational background, employment, work in the financial industry and monthly net income were also included.

### 2.2 Materials

Survey data were collected via an anonymous self-administered questionnaire. The questionnaire with the full phrasing of the surveyed items is included in Appendix A^1^.

### 2.3 Data collection

We randomly distributed the questionnaires to three types of financial clients between March and May in 2016 in Vietnam including: customers at stock brokerage companies and commercial banks and those who attended financial training workshops organized by the companies, as well as to students at universities or institutions of higher education (either full-time or part-time students while working). Additionally, people without financial educational backgrounds were recruited through “the quota and snow-ball sampling” survey method.

Of the 3144 respondents who completed the questionnaires, 448 were omitted due to too few answers (80% as a cutting point) or response differences in two pairs of identical items^2^. From those, 647 participants were individual investors who were trading in the Vietnamese stock market. However, we further excluded 26 participants due to their extreme answers on the four hypothetical choice tasks^3^. Therefore, our dataset includes only 621 records as identified in this data article.

## Footnotes

1 We only present the part of our original questionnaire used in Phan et al. [6]. The remaining part was reported in the study of Phan et al. [5]

2 In the original version of the questionnaire, we included 17 five-point-Likert-scale statements towards financial attitudes as proposed by Fünfgeld and Wang [3]. Two of them were repeated twice. Subjects were excluded from the dataset if providing different answers to those questions. The results regarding these statements can be found in Phan et al. [5].

3 Subjects are defined as outliers if their interest rates elicited from the hypothetical choice tasks fell out the 25–75% quantiles of the related data.

## References

[1] Dohmen T., Falk A., Huffman D., Sunde U., Schupp J., Wagner G.G. (2011). Individual risk attitudes: measurement, determinants, and behavioral consequences. J. Eur. Econ. Assoc..

[2] Dorn D., Huberman G. (2005). Talk and action: what individual investors say and what they do. Rev. Financ..

[3] Fünfgeld B., Wang M. (2009). Attitudes and behaviour in everyday finance: evidence from Switzerland. Int. J. Bank Mark..

[4] IMF, I. M. F. World economic outlook database, 2016. URL 〈http://www.imf.org〉.

[5] Phan C.T., Rieger M.O., Wang M. (2018). Segmentation of financial clients by attitudes and behavior: a comparison between Switzerland and Vietnam. Int. J. Bank Mark..

[6] Phan C.T., Rieger M.O., Wang M. (2018). What leads to overtrading and under-diverisfication? Survey evidence from retail investors in an emerging market. J. Behav. Exp. Financ..

[7] Sutter M., Kocher M.G., Glätzle-Rützler D., Trautmann S.T. (2013). Impatience and uncertainty: experimental decisions predict adolescents׳ field behavior. Am. Econ. Rev..

[8] Tangney J.P., Baumeister R.F., Boone A.L. (2004). High self-control predicts good adjustment, less pathology, better grades, and interpersonal success. J. Pers..

