# Supplementary material for: Survey data on Vietnamese retail investors׳ trading behavior and their psychological and behavioral patterns
Source: Data Brief. 2018 May 25;19:1176–80. doi: 10.1016/j.dib.2018.05.113 (PMC6140283; doi:10.1016/j.dib.2018.05.113)
Supplement: Application 2 [file mmc2.zip › Questionnaire DIB.pdf]

Questionnaire

In this questionnaire, we ask you some questions regarding your personal attitudes and behavior towards financial affairs.

This questionnaire is composed of three parts:

- In the first part, you are asked to rate how much personally you agree or disagree with statements in financial practices.
- In the second part, we ask you to make some hypothetical decisions in an experiment aiming at eliciting risk and time preferences.
- Finally, you are asked to provide some background information about yourself.

Remember there are no right or wrong answers.
We are interested in your own preferences and attitudes.

PART 1: ATTITUDES AND BEHAVIOR

A. To what extent do you agree or disagree with each of the following statements? *(Please mark one answer in each row with a circle.)*

	Strongly Agree	*Agree*	*Undecided*	*Disagree*	*Strongly disagree*
1. When I make plans, I am certain that they will work out.	1	2	3	4	5
2. I always know the status of my personal finances.	1	2	3	4	5
3. I am in control of my personal finances.	1	2	3	4	5
4. I'm controlling and am fully responsible for the results of my investment decisions.	1	2	3	4	5
5. My past investment successes were, above all, due to my specific skills.	1	2	3	4	5
6. My instinct has often helped me to make financially successful investments.	1	2	3	4	5

B. Please read the following 10 statements and for each, mark one answer that best represents you. *(Please mark one answer in each row with a circle.)*

	Very much like me	*Mostly like me*	*Somewhat like me*	*A little like me*	*Not at all like me*
1. I have a hard time breaking bad habits.	1	2	3	4	5
2. I get distracted easily.	1	2	3	4	5
3. I say inappropriate things.	1	2	3	4	5
4. I refuse things that are bad for me even if they are fun.	1	2	3	4	5
5. I am good at resisting temptation.	1	2	3	4	5
6. People would say that I have very strong self-discipline	1	2	3	4	5
7. Pleasure and fun sometimes keep me from getting work done.	1	2	3	4	5
8. I do things that feel good in the moment but regret later on.	1	2	3	4	5
9. Sometimes I can't stop myself from doing something, even if I know it is wrong.	1	2	3	4	5
10. I often act without thinking through all the alternatives.	1	2	3	4	5

C. In this question, we are interested in getting to know your personal forecasts for a stock index. For this purpose, we show you the historical price chart of VN–index (VNI) for five years.

Figure 1: VN–Index from 11/2011 to 11/01/2016

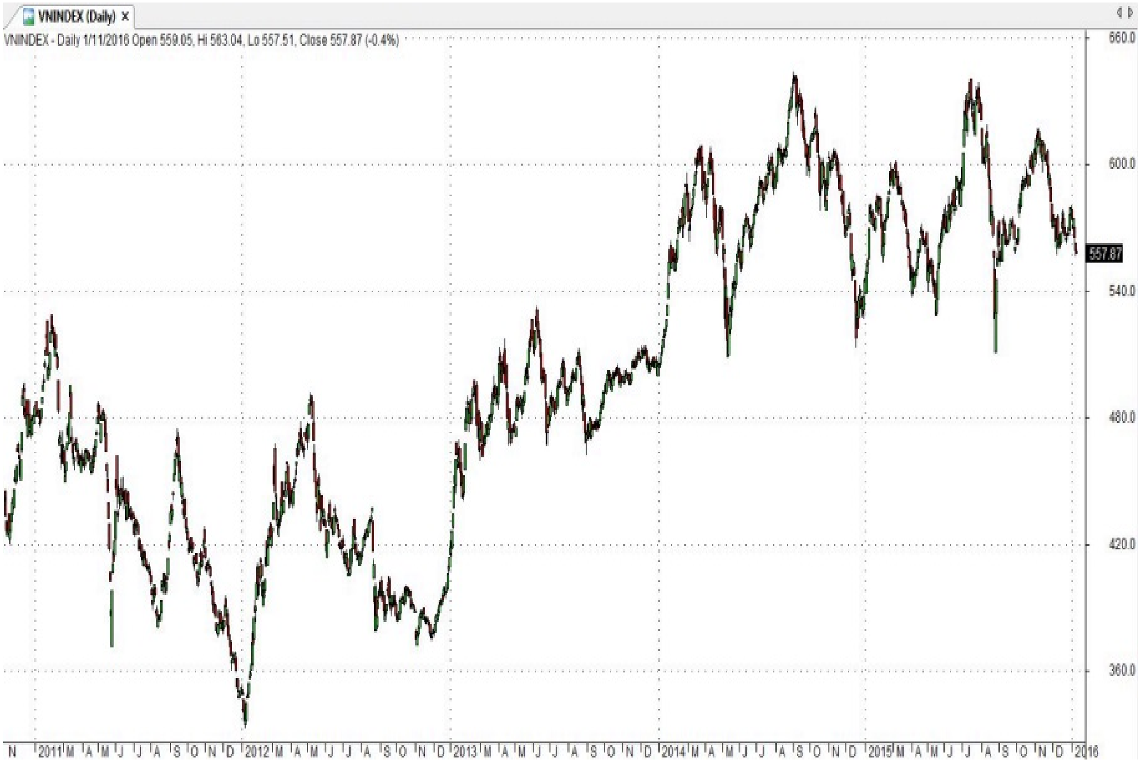

How many points could the price of the VN index stock reach in one year?

Please give the estimated **upper, average and lower bounds** by giving a number of points the VN–index could reach in **one year**.

Upper bound (will fall short in 19 out of 20 cases): ☐

Estimated average (will be in 10 out of 20 cases): ☐

Lower bound (will be exceeded in 19 out of 20 cases): ☐

Note that: An estimate of lower bound and upper bound in 19 out of 20 cases means the correct answers should be above your lower bound and lower the upper bound. Additionally, the estimated average bound in 10 out of 20 cases means that the correct answer is equally likely above or below your best guess.

D. How do you see yourself in the financial domain? Would you describe yourself as someone who tries to avoid risks (risk–averse) or as someone who is willing to take risks (risk–prone)?

Please tick a box on the scale, where the value 0 means: "risk averse" and the value 10 means "fully prepared to take risks" or "risk-prone". You can use the values in between to make your estimate.

Risk averse										Risk prone
0	1	2	3	4	5	6	7	8	9	10
⊙	⊙	⊙	⊙	⊙	⊙	⊙	⊙	⊙	⊙	⊙

PART 2: HYPOTHETICAL DECISIONS

In this part, you will be asked to choose between 2 alternatives: (1) an amount of money that can be received immediately and (2) alternative amount that can be received after a delay in different time horizons: **1 month** and **1 year**. Although you will not actually receive the rewards of money, please make each choice as if they were real without any risks associated with the delayed option. In other words, you are guaranteed to receive an amount of money after the specified delay.

1. Imagine that you have an opportunity to receive a payment of **2,000K VND** in **1 month from Today**. Please indicate at least the amount of payment **Today** in the box below so as to have an equally attractive alternative between **Today** and **1 month from Today**.

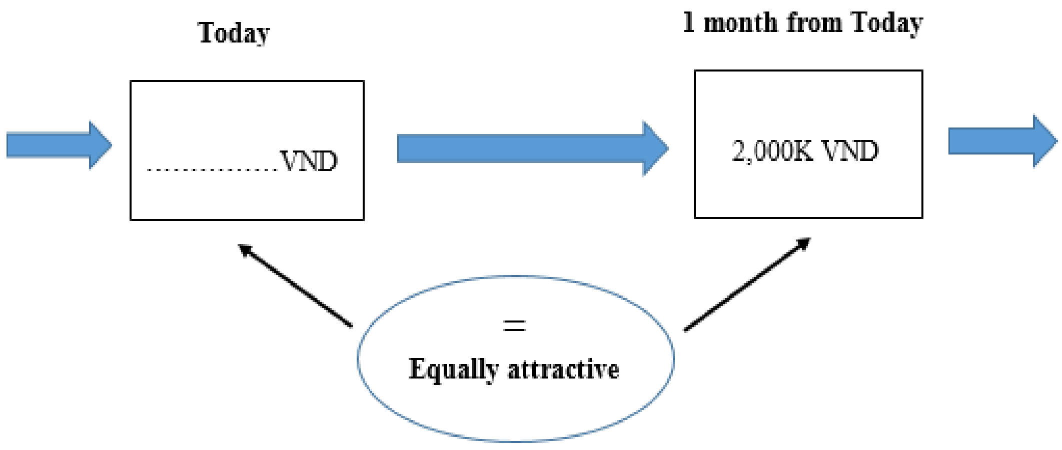

2. Imagine that you have an opportunity to receive a payment of **2,000K VND** in **2 months from Today**. Please indicate at least the amount of payment

in **1 month from Today** in the box below so as to have an equally attractive alternative between in **1 month** and **2 months from Today**.

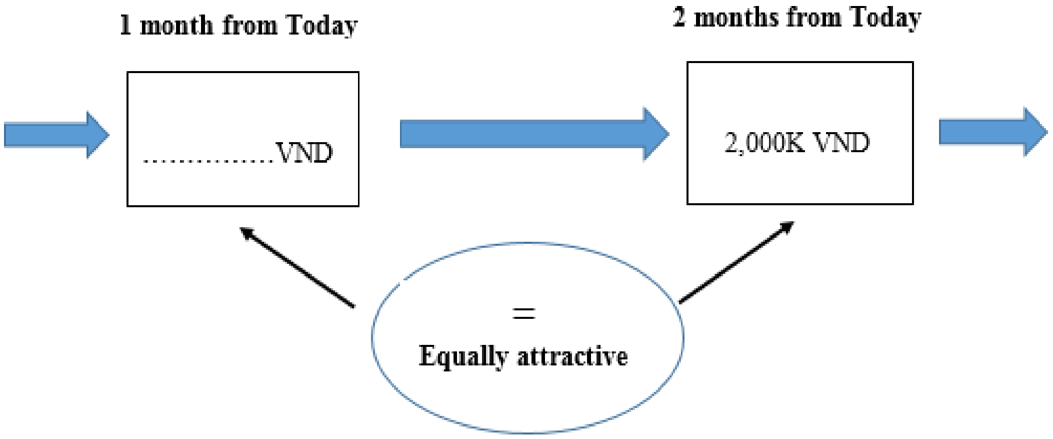

3. Imagine that you have an opportunity to receive a payment of **2,000K VND** in **1 year from Today**. Please indicate at least the amount of payment **Today** in the box below so as to have an equally attractive alternative between **Today** and **1 year from Today**.

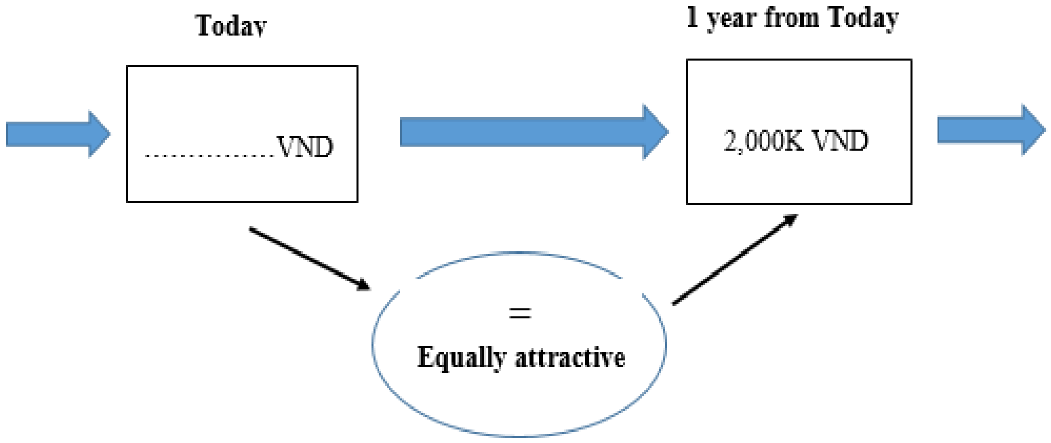

4. Imagine that you have an opportunity to receive a payment of **2,000K VND** in **13 months from Today**. Please indicate at least the amount of payment **1 month from Today** in the box below so as to have an equally attractive alternatives between in **1 month** and **13 months from Today**.

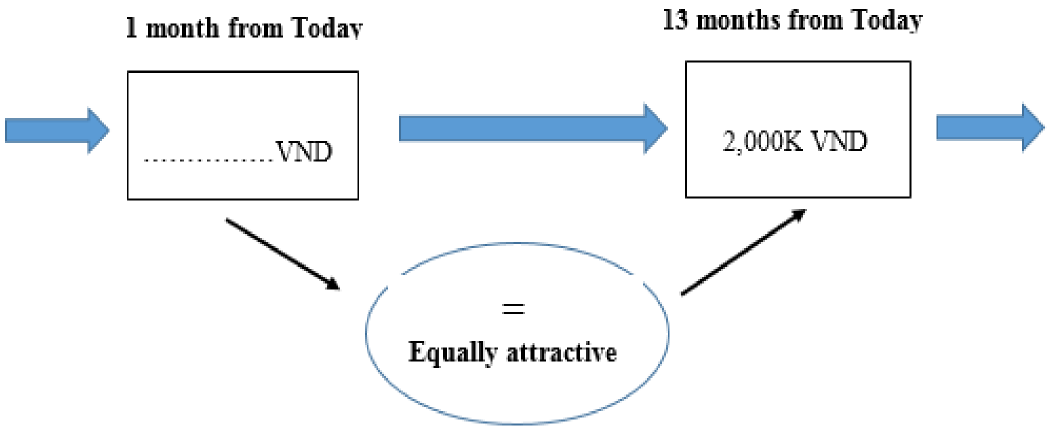

h

PART 3: SOCIO-DEMOGRAPHIC CHARACTERISTICS

Please answer the following questions about yourself. All answers are confidential and cannot be traced back to you personally.

A. STOCK INVESTORS

1. Have you ever invested in stocks or stock market funds? ⊙Yes ⊙No

If yes, would you please answer the following questions? Otherwise, continue with part B.

2. How much is your current investment portfolio on the Vietnamese stock market?

a. 50 Million VND or less
b. 50–100 Million VND
c. 100–250 Million VND
d. 250–500 Million VND
e. 500–1 Billion VND
f. 1–2 Billion VND
g. >2 Billion VND

3. How many times per month do you trade stocks?

a. 1–3 trades
b. 4–5 trades
c. 6–10 trades
d. 11–15 trades
e. >15 trades

4. How many times do you check your stock account?

a. More than 3 times per day
b. Daily
c. A couple of times per week
d. Weekly
e. Monthly
f. Quarterly
g. Annually

5. Have you currently invested in stock market funds? ⊙Yes ⊙No

If not, *how many different single stocks do you have currently in your investment portfolio? (If you are not entirely sure, please make a best guess).*

a. No stock
b. 1 stock
c. 2 stocks
d. 3–5 stocks
e. 6–10 stocks
f. More than 10 stocks

6. Why have you purchased stocks recently? *(Check all answers that apply)*

a. Work for company
b. Advice from friends and family
c. Advice from financial advisors
d. Launched good news of company (good financial reports, highly appreciated managers recruited. . .)
e. Stock price dropped considerably
f. Have expectations that you can earn more money on the stock market than with deposits
g. Others, please specify ________

7. Where do you get financial sources/information for stock investing activities? *(Check all answers that apply)*

a. At financial sections on websites of stock (brokerage) companies
b. At conferences/workshops organized by stock companies
c. At websites specialized in stock markets
d. Through the stock market analysis directly sent to stock investors
e. At some on-line forums for stock investors
f. Through personal contacts
g. Others, please specify ________

8. Why have you sold stocks recently? *(Check all answers that apply)*

a. Need cash
b. Poor performance of stock
c. Profit
d. Work for company
e. Reinvest
f. Poor management of company
g. Advice from financial advisors
h. Advice from friends and family
i. Reaching target/expected prices
j. Others, please specify ________

B. BACKGROUND INFORMATION

9. Are you male or female? ⊙Male ⊙Female

10. How old are you?

a. 18–35 years old
b. 36–50 years old
c. 51–55 years old
d. 56–65 years old
e. 66–85 years old
f. >85 years old

11. What **Highest** type of educational certificates/degrees have **YOU** received?

a. No school–leaving certificate
b. Elementary school certificate
c. Intermediate school certificate
d. High school certificate
e. Vocational school degree
f. College/Bachelor degree
g. Master degree
h. Doctor degree

12. Are you currently a student at university? ⊙Yes ⊙No

13. What is your **First** study major?

a. Economics, Finance or Business
b. Mathematics
c. Natural sciences
d. Medicine
e. Social sciences
f. Humanities
g. Arts
h. Others, please specify __________

14. What is your **Second** (if applicable) study major?

a. Economics, Finance or Business
b. Mathematics
c. Natural sciences
d. Medicine
e. Social sciences
f. Humanities
g. Arts
h. Others, please specify __________

15. Are you currently employed? ⊙Yes ⊙No

If **yes**, which one of the followings applies best to your occupational status?

a. Retired
b. Unemployed
c. Housewife
d. Self–employed
e. Part–time employed
f. Full–time employed
g. Others, please state __________

16. Are you currently working in financial sector? ⊙Yes ⊙No

17. What is your net income per month?

a. 5 Million VND or less
b. 5–10 Million VND
c. 10–20 Million VND
d. 20–50 Million VND
e. 50–100 Million VND
f. >100 Million VND

Thank you very much for your participation into the survey!
